# A comparative analysis of recurrence risk predictions in ER+/HER2− early breast cancer using NHS Nottingham Prognostic Index, PREDICT, and CanAssist Breast

**DOI:** 10.1007/s10549-022-06729-7

**Published:** 2022-09-10

**Authors:** Aparna Gunda, Mallikarjuna S. Eshwaraiah, Kiran Gangappa, Taranjot Kaur, Manjiri M. Bakre

**Affiliations:** OncoStem Diagnostics Pvt. Ltd., # 4 Raja Ram Mohan Roy Rd, Aanand Tower, 2nd Floor, Bangalore, 560 0027 India

**Keywords:** CanAssist Breast, Prognostication, Early breast cancer, Nottingham Prognostic Index, PREDICT

## Abstract

**Aims:**

Clinicians use multi-gene/biomarker prognostic tests and free online tools to optimize treatment in early ER+/HER2− breast cancer. Here we report the comparison of recurrence risk predictions by CanAssist Breast (CAB), Nottingham Prognostic Index (NPI), and PREDICT along with the differences in the performance of these tests across Indian and European cohorts.

**Methods:**

Current study used a retrospective cohort of 1474 patients from Europe, India, and USA. NPI risk groups were categorized into three prognostic groups, good (GPG-NPI index ≤ 3.4) moderate (MPG 3.41–5.4), and poor (PPG  > 5.4). Patients with chemotherapy benefit of < 2% were low-risk and ≥ 2% high-risk by PREDICT. We assessed the agreement between the CAB and NPI/PREDICT risk groups by kappa coefficient.

**Results:**

Risk proportions generated by all tools were: CAB low:high 74:26; NPI good:moderate:poor prognostic group- 38:55:7; PREDICT low:high 63:37. Overall, there was a fair agreement between CAB and NPI[κ = 0.31(0.278–0.346)]/PREDICT [κ = 0.398 (0.35–0.446)], with a concordance of 97%/88% between CAB and NPI/PREDICT low-risk categories. 65% of NPI-MPG patients were called low-risk by CAB. From PREDICT high-risk patients CAB segregated 51% as low-risk, thus preventing over-treatment in these patients. In cohorts (European) with a higher number of T1N0 patients, NPI/PREDICT segregated more as LR compared to CAB, suggesting that T1N0 patients with aggressive biology are missed out by online tools but not by the CAB.

**Conclusion:**

Data shows the use of CAB in early breast cancer overall and specifically in NPI-MPG and PREDICT high-risk patients for making accurate decisions on chemotherapy use. CAB provided unbiased risk stratification across cohorts of various geographies with minimal impact by clinical parameters.

**Supplementary Information:**

The online version contains supplementary material available at 10.1007/s10549-022-06729-7.

## Introduction

Not all patients diagnosed with early-stage ER+/HER2 neu negative breast cancer are required to be treated with adjuvant chemotherapy [1]. Oncologists rely upon clinical and pathological parameters such as tumor size, node status, tumor grade, and menopausal status, to assess the risk of cancer recurrence and accordingly determine the need for chemotherapy [1, 2]. Over time, computational algorithms, and mathematical equations have been developed using the clinical outcome data of various cancer registries. [3, 4]. Nottingham prognostic index (NPI), Adjuvant, NHS PREDICT, IHC4, and CancerMath are the widely used freely available prognostic tools for ER+/HER2 neu negative early breast cancer patients [5–10].

NPI is an established prognostic tool developed in the UK in 1982 that uses three clinical parameters to risk categorize the patients [11]. NHS PREDICT, on the other hand, is an online tool used to project the overall survival and disease outcomes with/without adjuvant chemotherapy that is developed on a cohort of patients in the U.K [6]. These online tools based on clinicopathological parameters provide doctors with an exact percentage of absolute chemotherapy benefit or survival estimates for each patient which helps in explaining treatment strategy to the patient. While these tools are useful to clinicians as they are free, and available online thus helping to prescribe treatment to patients without any delay, there are certain limitations. These online tools have been reported to underestimate or overestimate survival in patients of certain age groups. In patients under 40 years, PREDICT overestimates all-cause mortality by 8% [2, 12, 13]. This data raises the question of universal adaptability of these online tools which primarily function based on clinical parameters, age and proliferation markers. Moreover, relying on online tools which do not consider the deeper biology of the disease with an appropriate statistical approach may deprive patients of adequate treatment, particularly in those with small but biologically aggressive tumors who may benefit from chemotherapy [2, 13]. For a heterogeneous disease like breast cancer, a prognostic test that looks at the biology of the disease in depth beyond anatomy, proliferation, and hormonal indices along with clinical parameters and coupled with an AI/ML-based algorithm will certainly give the test an edge of high accuracy [14].

CanAssist Breast (CAB), a prognostic test developed based on Indian patients’ tumor tissues tries to balance all the factors mentioned above. CAB is a proteomics-based test that analyses the expression of 5 protein biomarkers using immunohistochemistry technique. This information coupled with the patient’s tumor-specific information (tumor size, grade, and node status) is used by a machine learning-based algorithm to compute the risk of cancer recurrence [15]. CAB segregates patients into two actionable risk groups, i.e., ‘low or high’ risk for cancer recurrence. CAB has been validated on more than 3500 patients across India, USA, and Europe and has been used prospectively in ~ 3000 patients to date to plan treatment since its launch [16–19].

The aims of the current report are multi-fold. Here we (i) compare the recurrence risk predictions of CAB with online tools, NPI and PREDICT, (ii) identify the sub-groups where CAB risk predictions imply precise treatment plan and finally, (iii) compare the risk predictions by these tools across different geographies to assess the influence of racial and ethnic differences, if any.

## Methods

### Patient samples/Data collection

This retrospective study included formalin-fixed paraffin-embedded (FFPE) tumor tissues from 1474 early-stage (stage I, II, and IIIA) hormone receptor-positive, HER2/neu negative consecutive breast cancer patients (with follow-up available) collected from hospitals and biorepositories in India (*n* = 473), USA (*n* = 137), and Europe (*n* = 864) diagnosed between 2007 and 2016. Primary surgical blocks of FFPE tumor specimens from these patients along with patient age, clinical parameters, and treatment follow-up details were obtained from the respective treating hospital or biorepository. These samples were part of the earlier CAB validation studies [16–18]. The samples were obtained with the IRB and ethics committee approvals of all the participating hospitals.

### CAB

Following haematoxylin & eosin staining to assess tumor quality, IHC was performed on consecutive sections for five CAB biomarkers (CD44, ABCC4, ABCC11, N-Cadherin, pan-Cadherin) on an automated Ventana platform [20, 21]. Post IHC grading by trained oncopathologists; this absolute percentage grading information for CD44, ABCC4, ABCC11, N-Cadherin, and pan-Cadherin, staining intensity for pan-Cadherin along with node status, tumor size, and tumor grade were used as inputs into the SVM—based algorithm to arrive at low-risk (CAB Risk Score ≤ 15.5) or high risk (CAB Risk Score > 15.5) category for each patient [15].

### NPI

NPI uses the following equation of clinical parameters to arrive at an index.

NPI = maximum invasive tumor size (S in cm) × 0.2 + lymph node stage (LN = 1, 2, or 3) + histological grade (*H* = 1, 2, or 3).

The NPI index generated six categories [11]. In our study, excellent and good prognostic groups were merged and called “Good Prognostic Group” (NPI score ≤ 3.4); moderate I and II prognostic groups (NPI score > 3.41 to ≤ 5.4) were merged and called “Moderate Prognostic Group” and the poor prognostic group I and II (NPI score ≥ 5.4) were merged and called “Poor Prognostic Group”.

### PREDICT

PREDICT V2 (https://breast.predict.nhs.uk/) is an online tool that predicts overall survival (OS) by providing a magnitude of benefit from chemotherapy over multiple time periods (5, 10, 15 years) using multiple parameters [22]. Based on the inputs selected, the tool gives a 5-year OS rate (%) including additional chemotherapy benefit (%). 2% (and above) difference in survival rates of patients treated with hormone therapy and 3^rd^ generation chemotherapy was considered as chemotherapy benefit. In this analysis patients with < 2% chemotherapy benefit was considered as low-risk (LR) and ≥ 2% as high-risk (HR).

### Statistical analysis

Rates of recurrence at a distant site along with SE (standard error) are computed from Kaplan–Meier survival curves. Cox proportional hazards model has been used to compute hazard ratios. Concordance between CAB, NPI, and PREDICT risk categories were analyzed by kappa coefficient [23]. For kappa correlation NPI moderate and high-risk groups were combined. The *P*-value was double-sided and < 0.05 was considered significant.

## Results

### Description of the cohort

The study cohort included 1474 patients (Table 1). Thirty-three percent of the cohort were aged below or equal to 50 years and 67% were above 50 years. The median age of the cohort was 57 years (range 26–92). 

Fifty-four percent of the cohort had patients with T1 tumors and 45% had T2 tumors. Sixty-three percent of the cohort had node-negative tumors, 36.6% with N1 tumors, and 0.5% of the patients had N2 tumors. Sixty-three percent of the patients had G2 tumors, 23% were with poorly differentiated tumors (G3) and only 14% had G1 tumors. All patients were with ER-positive disease. Ki-67 data were available for 1159 patients, of which 66% of patients expressed Ki67 less than 14%. This cohort included patients from different geographical locations, Europe (59%), India (32%), and the USA (9%).

**Table 1:** Patient demographics of the study cohort

Clinicopathological feature	Subgroups	n (%)
	Total	1474 (100%)
Age of the patient at diagnosis	Age ≤ 40 years	105 (7%)
	Age ≤ 50 years	481 (33%)
	Age > 50 years	991 (67%)
Tumor size	T1 tumors (≥ 2 cm)	800 (54%)
	T2 tumors (2.1–5 cm)	663 (45%)
	T3 tumors (> 5 cm)	11 (1%)
Node status	N0	927 (63%)
	N1 (up to 3 nodes)	536 (36.5%)
	N2 (4–9 nodes)	7 (0.5%)
Histological grade	G1	199 (14%)
	G2	932 (63%)
	G3	343 (23%)
Ki-67	Low Ki67 (≤ 14%)	764 (66%)
	High Ki67 (> 14%)	395 (34%)

### Risk proportions comparison of CAB, NPI, and PREDICT in the total cohort

CAB stratified 74% of the cohort as low-risk (LR) and 26% as high-risk (HR) (Table 2). NPI classified 38% of the patients as Good + excellent prognostic group-(GPG) and 55% of the patients as Moderate prognostic I and II (MPG) and 7% were poor prognostic-(PPG). Whereas PREDICT classified 63% of the cohort as LR and only 37% of the patients as HR. We observed similar risk stratification by CAB across age groups. Whereas, with NPI and PREDICT, GPG and LR proportions increased with the age of the patient. LR proportions decreased with all the prognostic tools with an increase in tumor size, increase in the number of nodes with metastasis, and higher grade;  however the decrease was drastic for NPI and PREDICT. A tremendous drop was observed in NPI-GPG from 66.9% in T1 tumors to 3% in T2 tumors. Similarly, PREDICT showed a significant decrease in LR patients from 85% in T1 tumors to 37% in T2 tumors. However, with CAB the decrease in LR patients was not so drastic with 84% as LR in patients with T1 tumors and 64% in T2 tumors (Table 2). When we looked at the risk proportions in patients across grades, most of the patients with G1 tumors were called LR by all three tests. In G2 tumors, LR percentages were higher by CAB (83%) and PREDICT (72%) compared to NPI that identified only 41.6% as GPG. In patients with G3 tumors it is worthy to note CAB identified 40% as LR while PREDICT LR were 15%, whereas by NPI none of these patients were called GPG. It is notable to observe there were no PPG patients in N0, G1, and T1N0 sub-groups by NPI. It is interesting to see similar proportions of LR by CAB (91%) and PREDICT (92%) in the T1N0 sub-group although it was not the case in T1 and N0 sub-groups individually by each of these two tests, CAB and PREDICT (Table 2).

**Table 2:** Percentage of risk proportions by CanAssist Breast (CAB), NPI, and PREDICT in the total cohort and across various sub-groups

	CAB		NPI			PREDICT	
	Low-risk	High-risk	Good + Excellent prognostic group (GPG)	Moderate prognostic groups-I &II (MPG)	Poor prognostic groups (PPG)	Low-risk (CT benefit < 2%)	High-risk (CT benefit ≥ 2%)
Total cohort (*n* = 1474)	74	26	38	55	7	63	37
Age < 40 years (*n* = 105)	73	27	23	62	15	39	61
Age < 50 years (*n* = 481)	77	23	32	59	9	58	42
Age > 50 years (*n* = 991)	73	27	41	53	6	65	35
Age > 60 years (*n* = 587)	73	27	44	49	7	67	33
T1 tumors (*n* = 800)	84	16	66.9	33	0.1	85	15
T size 0–1 cm (*n* = 156)	91	9	82	18	0	96	4
T size 1.1–2 cm (*n* = 640)	82	18	63.9	36	0.1	82	18
T2 tumors (*n* = 663)	64	36	3	82	15	37	63
T size 2.1 to 3 cm (*n* = 484)	67	33	3	83	14	41	59
T size 3.1 to 4 cm (*n* = 140)	62	38	6	80	14	29	81
T3 tumors (*n* = 11)	9	91	0	82	8	0	100
N0 (*n* = 927)	86	14	56	44	0	79	21
N1 (*n* = 536)	56	44	6	76	18	35	65
One node positive (*n* = 362)	57	43	8	77	15	43	57
Two node positive(*n* = 118)	52	48	1.5	74.5	24	20	80
N2 (*n* = 7)	–	100	–	29	71	–	100
G1 (*n* = 199)	92	8	86	14	0	98.5	1.5
G2 (*n* = 932)	83	17	41.6	58	0.4	72	28
G3 (*n* = 343)	40	60	0	71	29	15	85
Low Ki67 (≤ 14%) (*n* = 764)	80	20	45	50	5	74	26
High Ki67 (> 14%) (*n* = 395)	69	31	31	60	9	50	50
Luminal-A like (*n* = 602)	81	19	50	48	2	78	22
Luminal-B like (*n* = 606)	72	28	29	62	9	52	48
T1N0 (*n* = 590)	91	9	85	15	0	92	8

A similar trend of risk stratification is observed even in node-based sub-groups. Of the three tests, LR patients were higher by CAB (86%) and PREDICT (79%) compared to NPI (56%) in node-negative patients. In the N1 sub-group, LR patients were higher by CAB (56%) followed by PREDICT (35%) and they were very low by NPI (6%) (Table 2).

### Distant event rates in various risk groups

The distant event rates within five years of breast cancer diagnosis remained less than 10% in the LR group/GPG of all three tests (Table 3). Although, the hazard ratio (HR) in the total cohort was significant by all tests, in the clinical high-risk sub-groups (T2/N1/G3 tumors) HR by at least one test or both tests (NPI and PREDICT) was not significant (Table 3). Unlike these two tests, CAB had a significant HR in all the sub-groups stratified by clinical parameters (Table 3).

**Table 3:** Distant event rates within five years of diagnosis by CanAssist Breast, NPI, and PREDICT

	CanAssist Breast			NPI				PREDICT		
	Distant event rates in Low-risk (% ± SE)	Distant event rates in High-risk (% ± SE)	Hazard ratio (95% CI), P-value	GPG (% ± SE)	MPG (% ± SE)	PPG (% ± SE)	Hazard ratio (95% CI), P-value	Low-risk (% ± SE)	High-risk (% ± SE)	Hazard ratio (95% CI), P-value
Total cohort	5.036 ± 0.66	13.55 ± 1.76	2.8 (1.8–4.35), < 0.0001	2.50 ± 0.67	9.20 ± 1	17 ± 3.69	*4.15 (2.81–6.14), < 0.0001	4.36 ± 0.67	11.99 ± 1.38	2.86 (1.93–4.24), < 0.0001
T1 tumors	3.93 ± 0.86	10.89 ± 3.25	2.86 (1.05–7.8), 0.0046	2.64 ± 0.79	9.85 ± 2.2	NA	3.86 (1.74–8.55), 0.0001	3.63 ± 0.88	14.29 ± 3.99	4.17 (1.4–12,4), < 0.0001
T2 tumors	8.28 ± 1.34	16.11 ± 2.39	2.026 (1.25–3.27), 0.0021	4.76 ± 4.65	10.21 ± 1.30	17.17 ± 3.8	*2.44 (0.67–8.8),0.36	8.19 ± 1.75	12.78 ± 1.64	1.59 (0.99–2.5), 0.07
N0	4.18 ± 0.71	14.31 ± 3	3.59 (1.63–7.9), < 0.0001	2.31 ± 0.66	9.95 ± 1.5	NA	4.46 (2.56–7.73), < 0.0001	4.2 ± 0.74	11.44 ± 2.3	2.883 (1.47–5.66), < 0.0001
N1	7.23 ± 1.49	13.13 ± 2.1	1.85 (1.08–3.16), 0.024	5.56 ± 3.82	8.57 ± 1.34	16.67 ± 3.7	*1.88 (0.65–5.4), 0.37	5.23 ± 1.63	11.74 ± 1.74	2.34 (1.37–4.17), 0.012
G1	3.26 ± 1.31	13.33 ± 8.78	4.36 (0.29–64), 0.048	2.34 ± 1.16	14.29 ± 6.61	NA	6.63 (0.85–51), 0.002	3.57 ± 1.33	33.3 ± 27.2	10.51 (0.025–4346), 0.0061
G2	5.31 ± 0.81	9.66 ± 2.37	1.85 (0.92–3.8), 0.038	2.6 ± 0.81	8.54 ± 1.2	0	3.35 (1.97–5.7), 0.0002	4.32 ± 0.79	10.52 ± 1.92	2.51 (1.39–4.52), 0.0004
G3	5.87 ± 2	16.5 ± 2.6	2.97 (1.61–5.5), 0.0036	NA	10.25 ± 1.94	17.35 ± 3.83	1.77 (0.9–3.4), 0.064	7.77 ± 3.73	13.08 ± 1.99	1.74 (0.75–5.4), 0.28

*In sub-groups where NPI has three risk categories, the hazard ratio is computed for GPG vs MPG + PPG

### Restratification of NPI and PREDICT risk groups by CAB

We observed a good agreement (97%) between CAB-LR and NPI-GPG in the total cohort. Only 3% of the NPI-GPG were called HR by CAB. 65% of the MPG and 35% PPG group were identified as LR by CAB. Even between PREDICT-LR and CAB-LR, a good agreement of 88% was observed in the total cohort. 12% of the PREDICT-LR were segregated as CAB HR and at the same time, 51% of the PREDICT HR were classified as LR by CAB. Good agreement (> 96%) was seen across all the age groups between CAB-LR and PREDICT LR/NPI-GPG. Across various clinical sub-groups, a substantial number of NPI-MPG, NPI-PPG, and PREDICT-HR were called LR by CAB including G3 tumors where all of them were PPG by NPI (Table 4).

**Table 4:** Restratification of NPI and PREDICT risk groups by CAB

		NPI			PREDICT	
		GPG	MPG	PPG	Low-risk (CT benefit < 2%)	High-risk (CT benefit ≥ 2%)
Total cohort (*n* = 1474)	CAB LR	97	65	25	88	51
	CAB HR	3	35	75	12	49
	PREDICT LR	99.6	45	–	–	–
	PREDICT HR	0.4	55	100	–	–
Age ≤ 40 years (*n* = 105)	CAB LR	100	72	37	93	61
	CAB HR	–	28	63	7	39
Age ≤50 years (*n* = 481)	CAB LR	100	70	39	90	58
	CAB HR	–	30	61	10	42
Age > 50 years (*n* = 991)	CAB LR	97	62	16	88	47
	CAB HR	3	38	84	12	53
Age > 60 years (*n* = 587)	CAB LR	96	62	3	88	42
	CAB HR	4	38	97	12	58
T1 tumors (*n* = 796)	CAB LR	98	56	–	91	46
	CAB HR	2	44	1	9	54
T2 tumors (*n* = 661)	CAB LR	95	70	26	82	54
	CAB HR	5	30	74	18	46
N0 (*n* = 927)	CAB LR	98	69	–	93	57
	CAB HR	2	31	–	7	43
N1 (*n* = 536)	CAB LR	85	61	27	70	49
	CAB HR	15	39	63	30	51
G1 (*n* = 199)	CAB LR	95	79	–	93	33
	CAB HR	5	21	–	7	67
G2 (*n* = 932)	CAB LR	99	73	–	90	66
	CAB HR	1	27	100	10	34
G3 (*n* = 343)	CAB LR	–	45	26	52	38
	CAB HR	–	55	72	48	62
Low Ki67 (≤ 14%) (*n* = 764)	CAB LR	98	68	42	89	55
	CAB HR	2	32	58	11	45
High Ki67 (> 14%) (*n* = 395)	CAB LR	95	64	16	88	50
	CAB HR	5	36	84	12	50
Luminal-A (*n* = 602)	CAB LR	98	66	39	89	52
	CAB HR	2	34	61	11	48
Luminal-B (*n* = 602)	CAB LR	96	67	42	88	54
	CAB HR	4	33	58	12	46
T1N0(*n* = 590)	CAB LR	98	66	–	95	50
	CAB HR	2	34	–	5	50

### Concordance of CAB risk with NPI and PREDICT

For the kappa correlation analysis, NPI-MPG and PPG were combined and considered high-risk. As observed by kappa correlation there was a fair agreement between CAB and NPI [0.31 (95% CI 0.278–0.376)] and with CAB and PREDICT [0.398 (95% CI 0.35–0.446)]. Moderate agreement was observed between CAB and PREDICT in sub-groups of patients aged above 50 years [0.429 (0.369–0.488)], 60 years [0.484 (0.408–0.560)], and N0 patient [0.406 (0.332–0.481)] sub-groups. However, a moderate agreement was observed between CAB and NPI only in a subgroup of patients with T1 tumors [0.478 (0.414–0.543)] (Table 5). Even across NPI and PREDICT there was only moderate agreement in the total cohort [0.529 (0.492–0.566)] (Additional Table 1).

**Table 5:** Kappa correlation analysis between CAB and NPI/PREDICT risk groups

	CAB vs NPI (moderate and poor prognostic groups are combined) *	95% CI	CAB vs PREDICT	95% CI
Total Cohort	0.31	0.278–0.346	0.398	0.35–0.446
Age ≤ 40 years	0.194	0.104–0.284	0.274	0.138–0.409
Age ≤ 50 years	0.340	0.260–0.420	0.245	0.196–0.294
Age > 50 years	0.350	0.306–0.395	0.429	0.369- 0.488
Age > 60 years	0.386	0.326–0.446	0.484	0.408–0.560
T1 tumors	0.478	0.414–0.543	0.446	0.362–0.531
T2 tumors	0.031	0.014–0.047	0.239	0.177–0.301
N0 tumors	0.312	0.262–0.362	0.406	0.332–0.481
N1 tumors	0.066	0.032–0.101	0.186	0.112–0.261
G1 tumors	0.200	0.016–0.384	0.202	0.047–0.451
G2 tumors	0.230	0.192–0.268	0.267	0.199–0.335
Luminal A	0.329	0.271–0.388	0.394	0.305- 0.483
Luminal B	0.243	0.196–0.291	0.351	0.283–0.420
Low Ki67	0.299	0.250–0.347	0.365	0.289–0.441
High Ki67	0.283	0.218–0.347	0.384	0.300–0.468

^*^For kappa correlation NPI moderate and poor prognostic groups were clubbed together and considered High risk (HR)

### Comparison of risk stratification of CAB, NPI, and PREDICT across geographies:

The demographics of Indian and European cohorts are presented independently in Additional Table 2. More than half of the Indian cohort had patients with T2 (76%) and N1 (52.4%) tumors. The European and USA cohorts were dominated by patients with T1 (69.6 and 67%) and N0 tumors (70.5 and 74%). The median tumor size in the European cohort was 1.6 cm (range 0.2–5.4), whereas it was 2.5 cm in the Indian cohort (range 0.9–8). In the Indian, European, and USA cohorts CAB LR proportions were 68%, 77%, and 77%, respectively. Whereas GGPs by NPI were 11% in Indian cohort but they were 50% and 55% in European and USA cohorts, respectively. Likewise, LR proportions by PREDICT were 35% in Indian and they were above 70% in European and USA cohorts (Table 6).

**Table 6:** Comparison of  risk proportions by CAB, NPI, and PREDICT across different cohorts

	CAB		NPI			PREDICT	
	Low-risk, *n* (%)	High-risk, *n* (%)	GPG, *n* (%)	MPG, *n* (%)	PPG, *n* (%)	Low-risk, *n* (%)	High-risk, *n* (%)
India	322(68%)	151 (32%)	52 (11%)	356 (75%)	65 (14%)	164 (35%)	309 (65%)
Europe	669 (77%)	195 (23%)	430 (50%)	401 (46%)	33 (4%)	660 (76%)	204 (24%)
USA	106 (77%)	31 (23%)	76 (55%)	57 (42%)	4 (3%)	98 (72%)	39 (28%)

Across the sub-groups of these two diverse cohorts, European and Indian, risk proportions by CAB were similar or with fewer differences, while risk proportions by NPI/PREDICT differed greatly (Tables 7, 8). In the European sub-cohort with an increase in tumor size from T1 to T2, the CAB LR decreased from 85 to 61% (Table 7) and in the Indian sub-cohort, they almost remained constant at 82% (T1) and 83% (T2) (Table 8). Whereas with NPI, in the European and Indian sub-cohorts, NPI-GPG were very low, at 4% and 3% in the T2 sub-group while in the T1 sub-group they were tremendously higher at 85% (European) and 41% (Indian). A similar trend was observed with PREDICT with respect to LR proportions in T1 and T2 sub-groups across both cohorts (Tables 7, 8). Even across the other clinical parameters (node status and histological grade); increased low-risk proportions by NPI and PREDICT correlated with clinical risk parameters reflective of low clinical risk (e.g., N0 versus N1 or G1 versus G3 tumors) while CAB risk stratification seems to be independent of this.

**Table 7:** Percentage of risk proportions by CAB, NPI, and PREDICT across various sub-groups in the European cohort

	CAB		NPI			PREDICT	
	Low-risk	High-risk	GPG	MPG	PPG	Low-risk (CT benefit < 2%)	High-risk (CT benefit ≥ 2%)
Age ≤ 40 years (*n* = 43)	84	16	49	49	2	67	33
Age ≤ 50 years (*n* = 249)	80	20	48	48	4	76	24
Age > 50 years (*n* = 615)	76	24	50	46	4	77	23
Age > 60 years (*n* = 392)	74	26	49	45	6	74	26
Median tumor size	1.6 cm						
T1 tumors (*n* = 600)	85	15	70	30	0	87	13
T size 0-1 cm (*n* = 113)	93	7	84	16	0	99	1
T size 1.1–2 cm (*n* = 487)	83	17	67	33	0	85	15
T2 tumors (*n* = 263)	61	39	4	83	13	52	48
T size 2.1 to 3 cm (*n* = 216)	61	39	4	85	11	56	44
T size 3.1 to 4 cm (*n* = 37)	54	36	5	81	14	35	65
N0 (*n* = 609)	89	11	66	34	0	88	12
N1 (*n* = 252)	51	49	10	77	13	48	52
one node positive (*n* = 168)	52	48	14	75	11	60	40
Two node positive (*n* = 55)	49	51	2	82	16	25	75
G1 (*n* = 137)	97	3	96	4	0	97	3
G2 (*n* = 573)	88	12	52	48	0	85	15
G3 (*n* = 154)	21	79	0	79	21	23	77
Low Ki67 (≤ 14%) (*n* = 561)	81	19	54	45	1	83	17
High Ki67 (> 14%) (*n* = 191)	66	34	31	60	9	53	47
Luminal-A like (*n* = 484)	81	19	55	43.5	1.5	84	16
Luminal-B like (*n* = 276)	69	31	36	57	7	60	40

**Table 8:** Percentage of risk proportions by CAB, NPI, and PREDICT across various sub-groups in the Indian cohort

	CAB		NPI			PREDICT	
	Low-risk	High-risk	GPG	MPG	PPG	Low-risk	High-risk
Age≤ 40 years (*n* = 57)	65	35	4	70	26	18	82
Age≤ 50 years (*n* = 193)	74	26	8	76	16	34	66
Age > 50 years (*n* = 278)	64	36	13	75	12	35	65
Age > 60 years (*n* = 137)	66	34	17	72	11	27	61
Median tumor size	2.5 cm						
T1 tumors (*n* = 104)	82	18	41	58	1	65	35
T size 1.1–2 cm (*n* = 94)	81	19	40	59	1	63	37
T2 tumors (*n* = 359)	83	17	3	80	17	27	73
T size 2.1 to 3 cm (*n* = 235)	67	33	1	81	18	28	82
T size 3.1 to 4 cm (*n* = 99)	66	34	6	80	14	26	74
N0 (*n* = 217)	78	22	20	80	0	51	49
N1 (*n* = 248)	61	39	3	73	24	22	78
One node positive (*n* = 170)	62	38	3	79	18	25	75
Two node positive (*n* = 54)	52	48	2	63	35	17	83
G2 (*n* = 265)	73	27	13	85	1.5	44	56
G3 (*n* = 170)	58	42	0	64	36	8	92
Low Ki67 (≤ 14%) (*n* = 174)	79	21	14	72	14	64	36
High Ki67 (> 14%) (*n* = 111)	66	34	12	74	14	27	73
Luminal-A like (*n* = 99)	80	20	18	71	11	47	53
Luminal-B like (*n* = 225)	71	29	10	76	14	33	67

## Discussion

Prognostication in early breast cancer has evolved over time from the use of clinicopathological factors as standalone parameters for recurrence risk prediction to the recent multi-gene/biomarker tests/tools with or without the inclusion of clinical parameters to predict accurate prognosis. Online tools although having proved to be valuable, are known to overestimate/underestimate survival and chemotherapy benefit in patients of certain age groups and races [12, 24–29]. With this data, the use of these online tools appears to be limited to certain groups of patients. Moreover, breast cancer being a heterogeneous disease has different disease outcomes across different races and ethnicities [30–33]. Use of prognostic test/tool developed on one cohort might have variable performance on a cohort that’s racially and ethnically different from its development cohort. On the other hand, the use of multi-gene tests in all patients across the globe is not plausible due to their costs. At this juncture of online tools and expensive multi-gene tests, CAB serves as a cost-efficient proteomics-based test with performance comparable to that of these multi-gene tests (Oncotype DX) [34]. In the current report, we compared the risk stratification by NPI and PREDICT with that of CAB to identify the clinical risk groups where CAB risk predictions could be more useful for planning therapy to the patients.

A recurrence risk assessment by NPI and PREDICT is primarily driven by the clinical parameters whereas tumor biology plays a critical role in recurrence risk assessment by CAB. There was a huge decrease in low-risk proportions with an increase in the clinical risk (increased tumor size, lymph node metastasis, and higher histological grade) with NPI/PREDICT as observed by a few NPI-GPG patients in patients with N1 or G3 orT2 tumors. Similarly, none of the patients with G1/N0 tumors (lower clinical risk) and few patients with T1 and G2 tumors belonged to the NPI-PPG category. A similar pattern was observed with PREDICT. This high correlation observed between risk proportions and clinical parameters for NPI/PREDICT was not seen with CAB, although one could see a moderate decrease (T1-84%, T2-64%) in low-risk patients with an increase in clinical risk from T1 to T2 and similarly in node-positive versus node-negative tumors. This performance of CAB with minimal influence by clinical parameters is because CAB gives more importance to tumor biology than clinicopathological factors for recurrence risk prediction [16–18]. Moreover, in the European and USA cohorts dominated by patients with clinically low-risk features (T1/N0 tumors) NPI and PREDICT low-risk patients were tremendously high. Indian cohort was very different from these two cohorts with a higher representation of clinically high-risk patients (patients with T2/N1 tumors) and therefore had decreased NPI-GPG and PREDICT low-risk proportions compared to European and USA cohorts. However, even in these divergent cohorts, with respect to clinical parameters, CAB risk proportions across these three cohorts, did not vary to the extent as observed with NPI/PREDICT. We have previously shown that CAB’s accuracy in predicting distant recurrence in the European cohort is similar to that of the Indian cohort [18]. This is because CAB assesses the risk of recurrence by assessing the expression of the proteins (CD44, ABCC4, ABCC11, N-Cadherin, pan-Cadherin) involved in critical signaling pathways involved in the invasion of blood vessels by tumor cells leading to the spread, and drug resistance pathways; other than hormone signaling (ER, PR, and associated genes) and cell division/cell death (MDM2, MELK, PTTG1, etc. [35–38] and is thus less influenced by the clinical parameters and racial differences as well.

Another interesting group where this phenomenon of dependency of NPI/PREDICT on clinical parameters is well exhibited is the sub-group that had a mixture of clinical low- and high-risk features (T1N0 with histological grade 3). There were 89 T1N0 patients with grade 3 tumors. All these patients were called MPG by NPI. Although CAB and PREDICT identified similar numbers as low-risk in this sub-group (45 by CAB and 43 by PREDICT), there was only 48% concordance in these low-risk patients between CAB and PREDICT (*k* = 0.011 (95% CI, 0.19 to 0.219). This low concordance depicts LR patients by CAB and PREDICT are different. With respect to clinical outcomes CAB had a higher distant metastasis-free survival (DMFS) (95.6%) compared to PREDICT (90.7%) demonstrating that CAB low-risk predictions correlate with better survival outcomes than PREDICT (data not shown). Published data shows that the CAB ‘low risk’ patients with T1N0 disease have an excellent DMFS of 98% [17]. Though NPI, PREDICT, and CAB risk prediction models all have tumor size, node status, tumor grade as common parameters, the utmost important feature present additionally in CAB which is missing in NPI/PREDICT is the analysis of tumor biology. This data once again re-iterates the importance of tumor biology in accurate risk stratification in these patients. This is critical for patient care as it helps to prevent not only over-treatment of patients but at times under treatment of ‘clinically low-risk’ patients.

Concordance in the low-risk groups between two prognostic tests boosts the confidence of the clinician in avoiding chemotherapy. CAB has a very good concordance with multi-gene prognostic tests with 83% of concordance between CAB and Oncotype DX and MammaPrint in the low-risk category [18, 28]. Here in the current report, it is notable to observe 97% concordance between CAB and NPI low-risk categories where the NPI low-risk proportions were just half of the CAB low-risk proportions. While with PREDICT, a concordance of 88% was observed in the low-risk category indicating that CAB helps in identifying patients requiring chemotherapy of the remaining 12% of PREDICT low-risk patients; additionally prevents overtreatment in PREDICT high-risk patients as 51% of PREDICT high-risk patients were called as low-risk by CAB. However, in the NPI-MPG where treatment decision-making is ambiguous, 65% were stratified as low-risk by CAB which was higher than PREDICT that stratified only 45% as low-risk (Table 3), with similar survival advantage by both CAB and PREDICT risk stratifications (survival rate improved by 1.7 in NPI-MPG-CAB low-risk group and 1.8 in NPI-MPG-PREDICT low-risk group [data not shown]). Thus, the use of CAB in the NPI-MPG helps patients plan therapy accurately. The NICE diagnostic guidelines recommend the use of multi-gene tests (Oncotype DX, Prosigna, and EndoPredict) in NPI-MPG patients where chemotherapy decisions are unclear, and the use of these multi-gene tests is shown to be an expensive affair [39, 40]. The committee does not recommend the use of IHC4 as it lacks analytical validity. Unlike IHC4, CAB though an immunohistochemistry-based test has undergone rigorous analytical validation proving the test is robust [20] along with extensive clinical validation [16–18]. There are also other equations available, Maggee score that works using the immunohistochemical gradings of ER, PR, HER2, Ki67 and clinical parameters and are known to have good concordance with Oncotype DX RS [41]. Recently we completed CAB validation in a DUTCH sub-cohort of patients who participated in the TEAM trial, randomized for hormone therapy regimens [42] and the manuscript is under review.

The strengths of the manuscript are the large cohort size of stage I and stage II breast cancer patients and patient cohorts from varied geographies. Limitations of the study include the lack of demonstration of the accuracy of CAB risk predictions in comparison with online tools in known patient cohorts who underwent treatment only as per NPI or PREDICT risk assessments. With respect to CAB, the limitation is the lack of data on a prospective cohort randomized for chemotherapy.

In summary, we conclude that CAB finds a greater number of low-risk patients, who could be spared chemotherapy compared to NPI and PREDICT due to the deep analysis of tumor biology it involves, over the online tools. In the NPI prognostic group with a greater than 3.4 index, CAB offered more precise prognostication than PREDICT. Moreover, in patients where treatment decisions are based on PREDICT, CAB could reduce over-treatment in many high-risk patients by stratifying them as low risk. Thus, we believe use of CAB is complementary to risk assessment by online tools and will help to make improved decisions to provide optimum treatment to breast cancer patients.

## Supplementary Information

Below is the link to the electronic supplementary material.

Supplementary file1 (DOCX 27 kb)

## Data Availability

The data analyzed during the current study was not available publicly as it contains patient information. The data generated in the current work based on patient information will be available from the corresponding author on reasonable request.
