# Supplementary material for: A comparative analysis of recurrence risk predictions in ER+/HER2− early breast cancer using NHS Nottingham Prognostic Index, PREDICT, and CanAssist Breast
Source: Breast Cancer Res Treat. 2022 Sep 10;196(2):299–310. doi: 10.1007/s10549-022-06729-7 (PMC9581859; doi:10.1007/s10549-022-06729-7)
Supplement: Supplementary file 1 — Supplementary file1 (DOCX 27 kb) [file 10549_2022_6729_MOESM1_ESM.docx]

|  | NPI vs PREDICT | |
| --- | --- | --- |
|  | kappa correlation coefficient | 95% CI |
| Total cohort | 0.53 | 0.49-0.57 |
| Under and equal to 40 years | 0.63 | 0.48- 0.78 |
| Under and equal to 50 years | 0.50 | 0.43- 0.56 |
| above 50 years | 0.54 | 0.49-0.59 |
| above 60 years | 0.56 | 0.50- 0.62 |
| T1 tumors | 0.53 | 0.47-0.59 |
| T2 tumors | 0.11 | 0.06- 0.15 |
| N0 tumors | 0.50 | 0.45-0.56 |
| N1 tumors | 0.22 | 0.16-0.29 |
| G1 tumors | 0.17 | 0.00- 0.34 |
| G2 tumors | 0.42 | 0.37- 0.46 |
| Luminal A | 0.43 | 0.37- 0.49 |
| Luminal B | 0.54 | 0.48- 0.60 |
| Low Ki67 | 0.44 | 0.39-0.49 |
| High Ki67 | 0.61 | 0.54-0.69 |

*CI: confidence Interval*

*Additional Table 1: Agreement between NPI and PREDICT by Kappa correlation coefficient*

|  |  | **Europe** | **India** | **USA** | **P-value** |
| --- | --- | --- | --- | --- | --- |
|  | **Total** | 864 | 473 | 137 |  |
|  |  | n (%) | n (%) | N (%) |  |
| **Age of the patient** | **Age <40 years** | 43 (5%) | 57 (12%) | 5 (4%) | <0.0001 |
|  | **Age<50 years** | 249 (29%) | 193 (41%) | 39 (28%) |  |
|  | **Age >50 years** | 616 (71%) | 278 (58%) | 98 (72%) |  |
| **Tumor size** | **T1 tumors** | 600 (69.5%) | 104 (22%) | 92 (67%) | <0.0001 |
|  | **T2 tumors** | 263 (30.4%) | 359 (76%) | 39 (23%) |  |
|  | **T3 tumors** | 1 (0.1%) | 10 (1%) | 0 |  |
| **node status** | **N0** | 609 (70.5%) | 217 (46%) | 101 (74%) | <0.0001 |
|  | **N1** | 252 (29%) | 248 (52.4%) | 36 (26%) |  |
|  | **N2** |  | 7 (1.5%) |  |  |
| **histological grade** | **G1** | 137 (16%) | 38 (8%) | 24 (17%) | <0.0001 |
|  | **G2** | 573 (66%) | 265 (56%) | 94 (69%) |  |
|  | **G3** | 154 (18%) | 170 (36%) | 19 (14%) |  |
| **Ki-67** | **Low Ki67 (<14%)** | 561 (75%) | 174 (61%) | 29 (24%) | <0.0001 |
|  | **High Ki67 (>14%)** | 191 (25%) | 111 (39%) | 93 (76%) |  |

*Additional Table 2: Distribution of clinical parameters across cohorts*
